# A Case of Adult Epiglottitis in a Patient With Uncontrolled Diabetes and Occupational Risks

**DOI:** 10.7759/cureus.27967

**Published:** 2022-08-13

**Authors:** Patil Balozian, Anastasiia Weiland, David Weiland, Danial Nasif, Lara Zakarna, Keyvan Ravakhah

**Affiliations:** 1 Internal Medicine, St. Vincent Charity Medical Center, Cleveland, USA; 2 Emergency Medicine, MetroHealth Medical Center and Cleveland Clinic Foundation, Cleveland, USA

**Keywords:** thickening of the epiglottis and aryepiglottic folds, smoking cessation, staphylococcus, streptococcus, infectious, uncontrolled diabetes mellitus, metal shavings, inhalation injury, airway compromise, acute epiglottitis in adults

## Abstract

Epiglottitis is inflammation of the epiglottis with or without the involvement of supraglottic structures including the hypopharynx. Timely diagnosis is crucial as the treatment of epiglottitis is tailored to the degree of airway obstruction. Most patients improve with conservative measures, while some require an emergent airway intervention. We report a case of a 39-year-old Caucasian male with a history of uncontrolled diabetes mellitus and smoking who presented to the emergency department (ED) with a sore throat, dry cough, odynophagia, and difficulty swallowing. He was afebrile, tachycardic, tachypneic, hypertensive, and saturating at 99% on room air. His physical examination was remarkable for drooling, muffled voice, pharyngeal swelling, and erythema. Laboratory tests were significant for leukocytosis, hyperglycemia, and hemoglobin A1c (HbA1c) of 14.3% with a negative infectious workup. Lateral neck X-ray and emergent direct fiberoptic laryngoscopy revealed findings of epiglottitis with airway patency. The patient did not require intubation and was started on intravenous dexamethasone, vancomycin, ampicillin-sulbactam, and humidified air with suctioning of secretions and quickly recovered. In addition to known risk factors for developing epiglottitis such as uncontrolled diabetes and smoking, our patient was exposed to metal shavings at his new job, an occupational hazard that might have contributed to his clinical presentation. Our case highlights the importance of a prompt diagnosis and risk factor identification in the management of epiglottitis in adults.

## Introduction

Epiglottitis is inflammation of the epiglottis with or without the involvement of supraglottic structures including the hypopharynx. The incidence of epiglottitis in the pediatric population has decreased in recent years likely owing to widespread *Haemophilus influenzae* type B vaccination, while the incidence in adults remains unchanged [1]. The differential diagnosis for epiglottitis includes benign conditions such as pharyngitis, laryngitis of bacterial or viral origin, and severe conditions causing airway obstruction that include angioedema, peritonsillar or retropharyngeal abscess, anaphylaxis, foreign body aspiration, and caustic ingestion [2]. Timely diagnosis is crucial as the treatment of epiglottitis is tailored to the degree of airway obstruction. Most patients improve with conservative measures, while 10%-18% require an emergent airway intervention [3,4]. Recognizing patient risk factors for developing epiglottitis with a high likelihood of airway compromise is therefore of great importance in successful management.

## Case presentation

**Figure 1:**
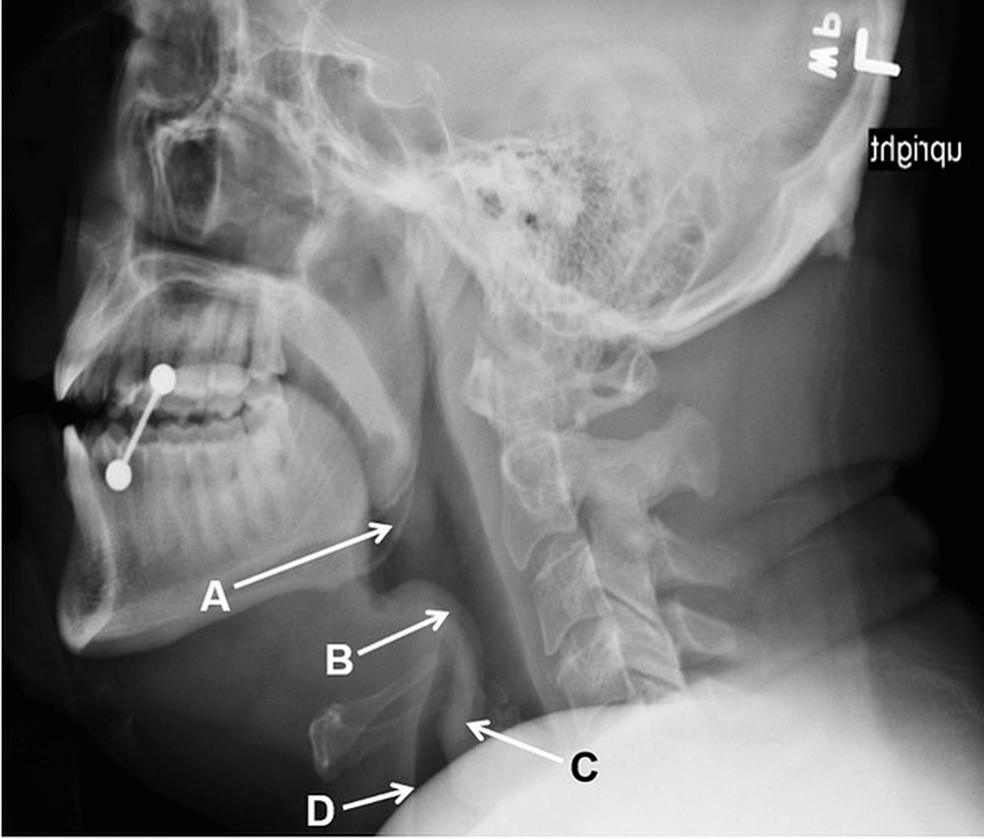
Lateral neck X-ray A: Thickening of the palatine tonsils. B: Thickening of the epiglottis. C: Thickening of the aryepiglottic folds. D: Patent airway.

A 39-year-old Caucasian male with a past medical history of uncontrolled diabetes mellitus type 2 (on glipizide and metformin), coronary artery disease (on metoprolol and aspirin), hypertension (on enalapril), hyperlipidemia (on atorvastatin and ezetimibe), smoking, and obesity class I with a body mass index (BMI) of 34 presented to the emergency department (ED) with a three-day history of sore throat, dry cough, and odynophagia. A day prior to his presentation, he was seen in an urgent care clinic where he tested negative for COVID-19 and was prescribed a course of amoxicillin-clavulanate that he was unable to take due to difficulty swallowing. Associated symptoms included myalgias. The patient denied alcohol or illicit drug use. He reported receiving all childhood vaccinations, flu vaccine, and COVID-19 vaccine with boosters. He had started a new job as a steel cutter; however, he had not been compliant with wearing a mask. In the ED, his heart rate was 131 bpm, blood pressure was 179/104 mmHg, temperature was 37.1°C, respiratory rate was 22 breaths/minute, and oxygen saturation was 99% breathing ambient air. His physical examination was significant for mild respiratory distress, tachycardia, drooling, muffled voice, pharyngeal swelling, and erythema, with the remainder of the examination being unremarkable. Laboratory tests were significant for a white blood cell count of 15,700 K/μL with a normal differential, glucose of 249 mg/dL, and hemoglobin A1c (HbA1c) of 14.3%, with a previous HbA1c of 9.5% only six months prior to his current presentation. Electrolytes, blood urea nitrogen, and creatinine were normal. Urinalysis was negative for ketones and beta-hydroxybutyrate. Repeat COVID-19 PCR, rapid influenza, and group A streptococcal antigen tests were negative. Two sets of blood cultures were obtained. Lateral neck X-ray showed thickening of the epiglottis and aryepiglottic folds with an enlargement of the palatine tonsils. The visualized airway appeared patent (Figure 1).

Emergent direct fiberoptic laryngoscopy revealed moderate swelling of the epiglottis and arytenoids, hooding of arytenoids with airway patency, mobile vocal cords bilaterally, and pooling of secretions. Intubation in the operating room performed by the anesthesia team was unsuccessful, which prompted an immediate patient transfer to a higher level of care. Upon arrival at the tertiary care center, the patient’s condition had slightly improved, and intubation was deferred. He was admitted to the ICU and was started on intravenous dexamethasone, vancomycin, ampicillin-sulbactam, humidified air, and suctioning of secretions as needed. On day 2 of hospitalization, enalapril was resumed as the patient did not have any lip or tongue swelling concerning angioedema secondary to angiotensin-converting enzyme (ACE) inhibitors. Vancomycin was also discontinued given his clinical improvement. Ampicillin-sulbactam was continued for four days. Blood cultures on day 4 showed no growth. He was initiated on insulin therapy, counseled on smoking cessation, and advised to wear a mask while at work.

## Discussion

Epiglottis in adults is a serious condition with a moderate to high risk of airway obstruction. Reported mortality varies greatly from 1% to 20% [5,6] likely due to limited availability of immediate expert evaluation by an otolaryngologist, anesthesiologist, or intensivist in some care settings. Nevertheless, a high degree of clinical suspicion by ED personnel plays an essential role in the outcome.

Moreover, it is important to promptly recognize risk factors associated with increased morbidity and mortality in adult epiglottitis. One large retrospective cohort study of more than 30,000 patients reported a strong association between older age, male sex, smoking status, and mortality on univariate analysis. However, on multivariate analysis, only the male sex was associated with higher mortality [5]. Diabetes is notoriously known to serve as a predisposing factor for infections due to impaired immunity and microvascular complications, leading to a compromised epithelial barrier. Increased incidence of epiglottitis in diabetics has been widely reported [7], as well as a twofold increased risk with higher two-day mortality in these patients [3,8]. There is a statistically significant association between epiglottitis and diabetes. It is due to the immune depression of polymorphonuclear leukocytes due to decreased leukocyte adhesion and phagocytic activity [7]. Hence, there could possibly be a correlation between epiglottitis and rapidly increasing HbA1c levels in patients with poorly controlled diabetes mellitus. Our patient’s previous HbA1c was 9.3%, which was increased to 14.3%, upon his current presentation. Furthermore, the statistically significant association between epiglottitis and male sex can be due to the role of androgens in the regulation of the immune system and lifestyle and socioeconomic factors [7].

While *Streptococcus* and *Staphylococcus *spp. are the most common identified causes of epiglottitis, diabetic patients may be affected by rare pathogens such as *Neisseria meningitidis* [9] and *Aeromonas hydrophila* [10]. Lastly, mucosal trauma caused by foreign body aspiration, caustic ingestions, and thermal burns due to inhalation of fumes or ingestion of hot substances has also been described [2,11].

Our patient had multiple risk factors, including tobacco use, a history of uncontrolled diabetes, and being of the male sex, predisposing him to epiglottitis and progression to asphyxia. However, his recent occupational exposure to metal shavings without the use of personal protective equipment may have also played a role. To our knowledge, this is the first report of adult epiglottitis in a steel cutter. It is possible that the inhalation of microscopic metal particles caused microtrauma of his upper airway mucosal lining. In our case, a diagnosis was made quickly, and the risk factors were promptly recognized by the caregivers, necessitating an intubation attempt in the operating room, although unsuccessful. Despite a failed intubation attempt, the patient recovered with conservative measures. A bacterial pathogen has not been identified in our patient, as in most cases of adult epiglottitis. Angiotensin-converting enzyme (ACE) inhibitor, which was initially discontinued upon the patient’s presentation, was resumed. The patient did not have signs concerning angioedema. The patient’s presentation was attributed to epiglottitis in the setting of occupational exposure and uncontrolled diabetes mellitus.

## Conclusions

Accurately identifying epiglottitis in adults is critical as an immediate airway intervention may be required; however, the majority of patients improve with conservative measures. Occupational risks, infectious triggers, and comorbidities including diabetes mellitus are the main risk factors for adult epiglottitis. Our case reflected the inhalation of metal shavings in steel cutters as a causative agent of epiglottitis in a patient with uncontrolled diabetes mellitus and a negative infectious workup. It is hence imperative to keep a high index of suspicion for the diagnosis of adult epiglottitis in a patient presenting with respiratory symptoms and overt physical examination findings in the setting of uncontrolled diabetes mellitus and occupational exposure. Upon discharge of adults with acute epiglottitis, it is primordial to address risk factor modification such as smoking cessation, diabetes control, and occupational hazard avoidance.
